# Glutathione-Based Photoaffinity Probe Identifies Caffeine as a Positive Allosteric Modulator of the Calcium-Sensing Receptor

**DOI:** 10.1021/acschembio.4c00335

**Published:** 2024-07-08

**Authors:** Nadee N. J. Matarage Don, Rayavarapu Padmavathi, Talan D. Khasro, Md. Rumman U. Zaman, Hai-Feng Ji, Jeffrey L. Ram, Young-Hoon Ahn

**Affiliations:** † Department of Chemistry, Drexel University, Philadelphia, Pennsylvania 19104, United States; ‡ Department of Physiology, Wayne State University, Detroit, Michigan 48201, United States

## Abstract

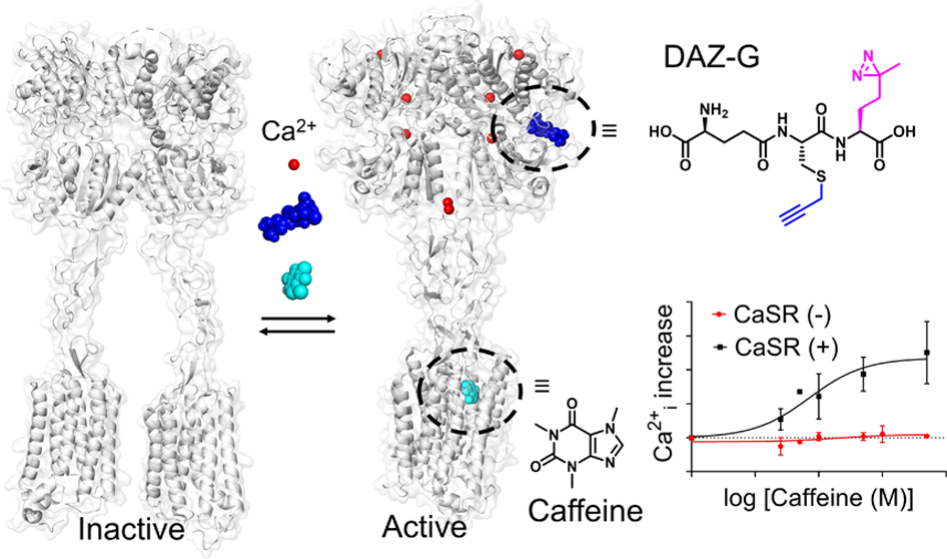

The calcium-sensing receptor (CaSR), abundantly expressed in the parathyroid gland and kidney, plays a central role in calcium homeostasis. In addition, CaSR exerts multimodal roles, including inflammation, muscle contraction, and bone remodeling, in other organs and tissues. The diverse functions of CaSR are mediated by many endogenous and exogenous ligands, including calcium, amino acids, glutathione, cinacalcet, and etelcalcetide, that have distinct binding sites in CaSR. However, strategies to evaluate ligand interactions with CaSR remain limited. Here, we developed a glutathione-based photoaffinity probe, DAZ-G, that analyzes ligand binding to CaSR. We showed that DAZ-G binds to the amino acid binding site in CaSR and acts as a positive allosteric modulator of CaSR. Oxidized and reduced glutathione and phenylalanine effectively compete with DAZ-G conjugation to CaSR, while calcium, cinacalcet, and etelcalcetide have cooperative effects. An unexpected finding was that caffeine effectively competes with DAZ-G′s conjugation to CaSR and acts as a positive allosteric modulator of CaSR. The effective concentration of caffeine for CaSR activation (<10 μM) is easily attainable in plasma by ordinary caffeine consumption. Our report demonstrates the utility of a new chemical probe for CaSR and discovers a new protein target of caffeine, suggesting that caffeine consumption can modulate the diverse functions of CaSR.

The calcium-sensing receptor (CaSR) is a class C G protein-coupled receptor (GPCR), abundantly expressed in the parathyroid gland and kidney, that plays a central role in calcium homeostasis.^1^ In parathyroid, CaSR negatively regulates parathyroid hormone (PTH) synthesis and secretion.^2^ PTH is a central hormone that stimulates calcium (Ca^2+^) reabsorption in the renal thick ascending limb, Ca^2+^ resorption in bone, and the synthesis of 1,25-dihydroxyvitamin D_3_ in the proximal renal tubule that induces Ca^2+^ absorption in the intestine.^3^ Therefore, high extracellular Ca^2+^ (Ca^2+^_o_)-mediated CaSR activation suppresses plasma PTH with a reduction in Ca^2+^_o_ level. In contrast, low Ca^2+^_o_-mediated CaSR inactivation increases plasma PTH with the restoration of Ca^2+^_o_ level.^1,2^ CaSR in the kidney also responds to the elevated Ca^2+^_o_ and suppresses Ca^2+^ reabsorption.^4^ The significance of CaSR for Ca^2+^ homeostasis is evidenced by the loss or gain of function mutations in CaSR, resulting in hypercalcemic and hypocalcemic disorders, respectively.^1^ In addition to Ca^2+^ homeostasis, CaSR in bone inhibits osteoclast-induced bone resorption and increases the proliferation of bone-forming osteoblasts, mediating bone growth and development.^5,6^ In addition, CaSR activation causes lung airway contraction, pulmonary remodeling, and fibrosis, which are associated with the development of asthma.^7^ Moreover, CaSR signaling is linked to NLRP3-mediated inflammatory rheumatoid arthritis and autoinflammatory disease,^8,9^ and the elevated levels of CaSR are linked to metastatic breast and prostate cancers,^10^ among others. Consequently, CaSR is a promising therapeutic target. CaSR’s positive allosteric modulators (PAM) (e.g., cinacalcet and etelcalcetide) are FDA-approved drugs to treat patients with secondary hyperparathyroidism.^11,12^ CaSR’s negative allosteric modulators (NAM), such as NPS2143, were evaluated for osteoporosis and are emerging therapeutics for asthma.^7,13,14^ Therefore, CaSR plays multifunctional roles in human physiology and pathology.

Structurally, CaSR forms a homodimer, each of which comprises the extracellular domain (ECD), 7-transmembrane (7TM) region, and intracellular C-terminal tail (Figure 1).^15^ The ECD includes the “Venus flytrap (VFT)” domain formed by two lobes (LB1 and LB2) and the cysteine-rich domain (CRD), which are connected to the 7TM and the C-terminal tail. Recent structural analyses elucidate complex and distinct conformations during ligand-mediated activation (Figure 1).^16−18^ In inactive CaSR, two protomers have minimal interactions between two LB1 regions while keeping the two protomers relatively apart, named an “open” state.^18^ In contrast, the active CaSR has significant interactions between the two protomers, where LB2, CRD, and 7TM in one protomer interact with their counterparts in another protomer while forming an asymmetrical twist between two protomers, called a “closed” state.^16^ However, multiple partially closed or open intermediate structures are thought to exist between two fully “open” and “closed” states.^17,18^

**Figure 1:**
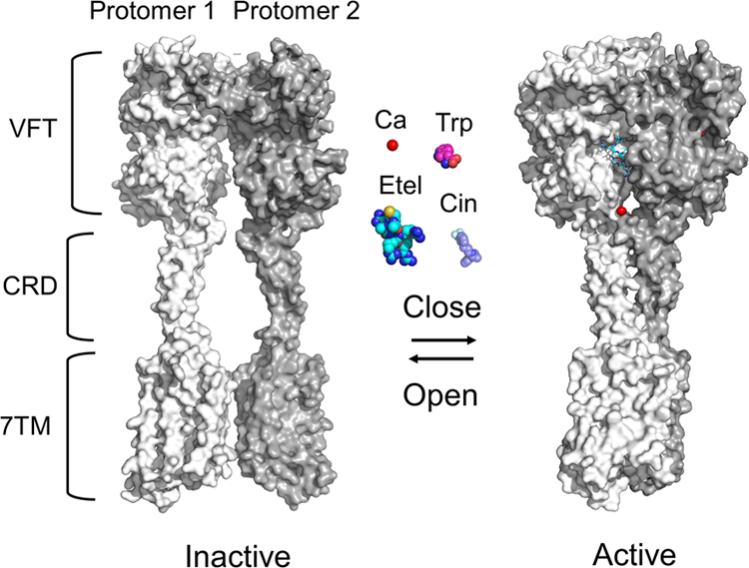
CaSR structure upon binding with its ligands. Each protomer comprises the Venus flytrap (VFT), cysteine-rich domain (CRD), transmembrane domain (7TM), and C-terminal tail. Without ligands, CaSR retains an inactive “open” state (left, PDB: 7M3J). When binding to its agonist (Ca^2+^_o_) or PAMs, such as l-Trp, cinacalcet (Cin), and etelcalcetide (Etel), CaSR turns to an active “closed” state (right, PDB: 7M3F).

Despite Ca^2+^_o_ being a nominal agonist, the interesting complexity is that the pleiotropic roles of CaSR are mediated by various endogenous agonists and allosteric modulators, along with multiple pharmacological drugs, that bind to several distinct binding sites in CaSR.^15^ The structural analyses support that Ca^2+^ binds to four sites in ECD (Ca^2+^ binding sites), some of which also bind to Mg^2+^.^19^ Amino acids, especially phenylalanine (Phe) and tryptophan (Trp), are PAMs, binding to the cleft between LB1 and LB2 (i.e., amino acid binding site, ABS).^15,20^ The ABS can accommodate larger PAM ligands, including γ-Glu-containing peptides, and reduced and oxidized glutathione (GSH and GSSG).^21−23^ Cinacalcet, the first FDA-approved CaSR PAM, binds to the pocket in 7TM (i.e., cinacalcet-binding site, CBS).^16^ Etelcalcetide, another FDA-approved CaSR PAM, binds between two LB2 (etelcalcetide-binding site, EBS) via a disulfide bond with C482 and salt bridges.^16,24^ In addition, CaSR senses anions (e.g., phosphate and Cl^–^) with a phosphate ion binding at ECD.^18,25^ Moreover, polyamine derivatives (e.g., spermine, spermidine, and histamine), pro-inflammatory and asthma inducers in the lung airway, are CaSR agonists without defined binding sites.^7,26^ Notably, many endogenous ligands and synthetic ligands regulate CaSR via binding to distinct binding sites while causing cell-type dependent and selective biased signaling among multiple downstream pathways.^15^ Therefore, identifying CaSR ligands and their modes of CaSR interactions is important to understand and control CaSR-mediated physiology and pathology.

Currently, CaSR ligands are discovered or validated mainly via CaSR’s downstream functional assays (e.g., Ca^2+^ flux) and radioactive ligand binding studies,^21,27^ which may limit the utility. Here, we developed a photoaffinity-tagged glutathione derivative (DAZ-G) as a chemical tool that enables the analysis of CaSR ligands. We demonstrate that DAZ-G binds to CaSR and acts as a PAM, elevating intracellular Ca^2+^ (Ca^2+^_i_) with relatively high potency (EC_50_ = 115 nM). We further demonstrate that DAZ-G enables the identification and analysis of CaSR ligands and their binding sites. Notably, DAZ-G was used to discover new ligands, finding that caffeine is a potent PAM for CaSR. Our data uncover a novel target protein of widely consumed caffeine, suggesting that caffeine’s pleiotropic effects could be partially mediated by its activation of CaSR. More broadly, the diverse physiological functions of CaSR in the human body may be modulated by daily consumed caffeine.

## Results and Discussion

### Glutathione-Based Photoaffinity Probe, DAZ-G, Labels CaSR

**Figure 2:**
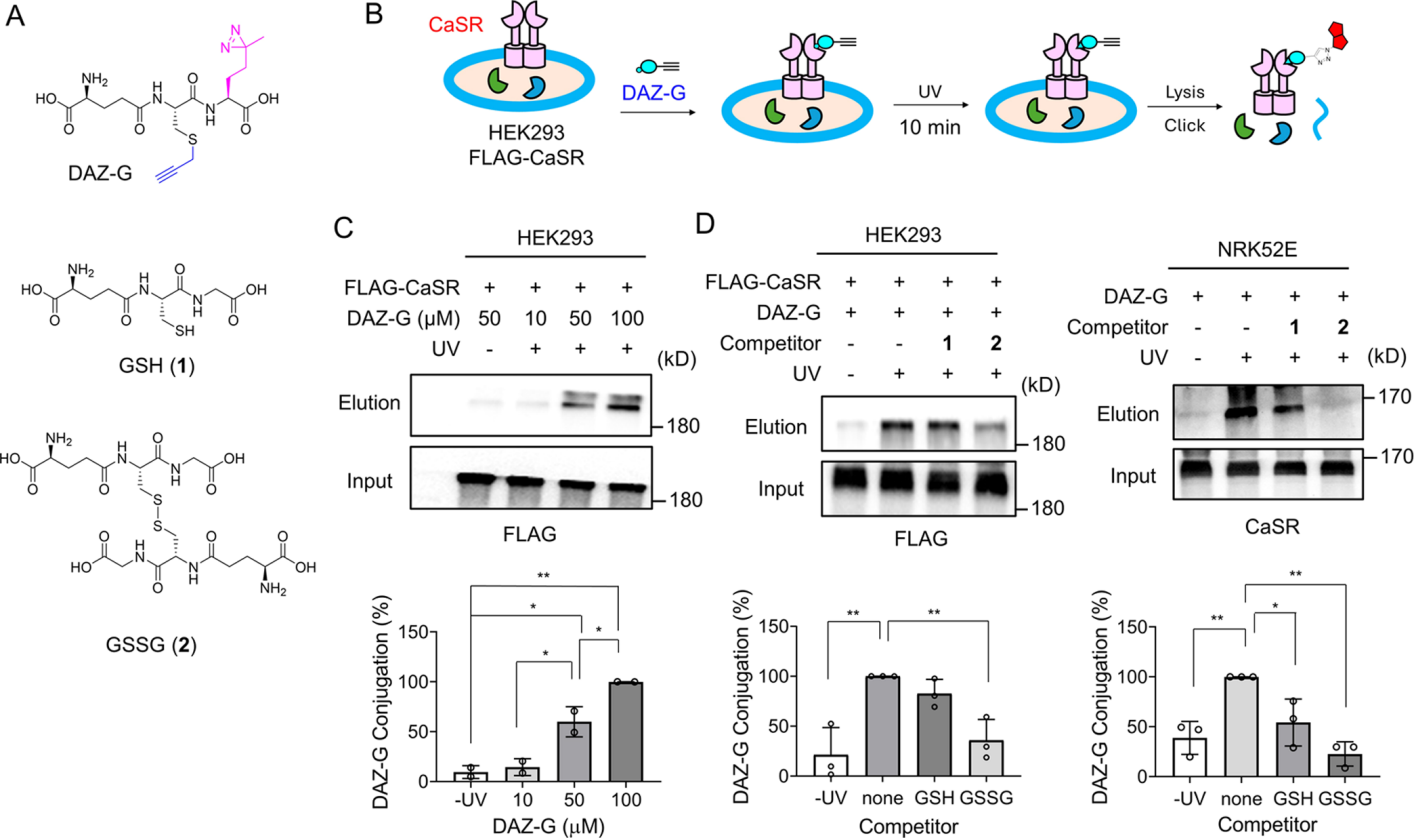
Photoaffinity-tagged GSH probe, DAZ-G, labels CaSR. (A) Structures of DAZ-G, GSH, and GSSG. (B) Strategy to monitor DAZ-G′s binding to CaSR. DAZ-G was incubated in cells expressing FLAG-CaSR. Cells were then irradiated with UV, inducing DAZ-G′s covalent conjugation to CaSR. After the click reaction of lysates with biotin-azide, biotinylated CaSR was analyzed by Western blots before (input) and after (elution) pull-downs with streptavidin–agarose. (C) DAZ-G conjugation to CaSR with increasing concentrations of DAZ-G. (D) DAZ-G conjugation to CaSR in the presence of each competitor [50 μM GSH (1) or GSSG (2)] in HEK293 ectopically expressing FLAG-CaSR (left) or NRK52E cells endogenously expressing CaSR (right). DAZ-G and competitors were added to cells in DMEM containing Ca^2+^_o_ (1.8 mM), Mg^2+^_o_ (0.8 mM), and 15 different amino acids. Data represent the mean ± SD with representatives from 3 independent experiments. The statistical difference was analyzed by one-way ANOVA and Dunnett’s *posthoc* test, where *p < 0.05, **p < 0.01, ***p < 0.001, and ****p < 0.0001.

To probe the binding events of ligands with CaSR, we thought of developing a photoaffinity-tagged CaSR ligand, especially one derived from GSH. Many γGlu-containing di- or tripeptides retain the binding specificity to the ABS in CaSR,^22,28^ suggesting that Cys and Gly positions in GSH could vary with modifications. Therefore, we designed a GSH-derived probe (DAZ-G) that contains photoaffinity diazirine and clickable alkyne groups at Gly and Cys positions in GSH (Figure 2A). DAZ-G was synthesized in two steps via coupling γGlu-Cys with a diazirine-containing Met analogue via our previously reported glutathione synthetase mutant (GS M4),^29,30^ followed by alkylation of propargyl group on the Cys (Figure S1).

Next, DAZ-G was evaluated to confirm its binding and conjugation to CaSR. Human CaSR was ectopically expressed in HEK293 cells (Figure S2A). After DAZ-G incubation, cells were irradiated for 10 min. Subsequently, lysates were analyzed after the click chemistry with biotin-azide (Figure 2B). The increasing concentration of DAZ-G showed increasing signals for DAZ-G conjugation to CaSR (Figures 2C and S2B), which was absent in cells without expression of CaSR (Figure S2B) or ultraviolet (UV) irradiation (Figure S2C). Importantly, DAZ-G conjugation to CaSR was competed by incubation of GSH or GSSG (Figures 2D, left, and S3A), supporting that DAZ-G binds to CaSR similarly to GSH and GSSG. Interestingly, GSSG showed more significant competition versus GSH, suggesting that GSSG may bind to CaSR stronger than GSH. Similarly, DAZ-G conjugation to CaSR and its competition by GSH or GSSG were observed in the NRK52E cell line expressing endogenous CaSR (Figures 2D, right, and S2A).

### DAZ-G Binds to the Amino Acid Binding Site in CaSR

**Figure 3:**
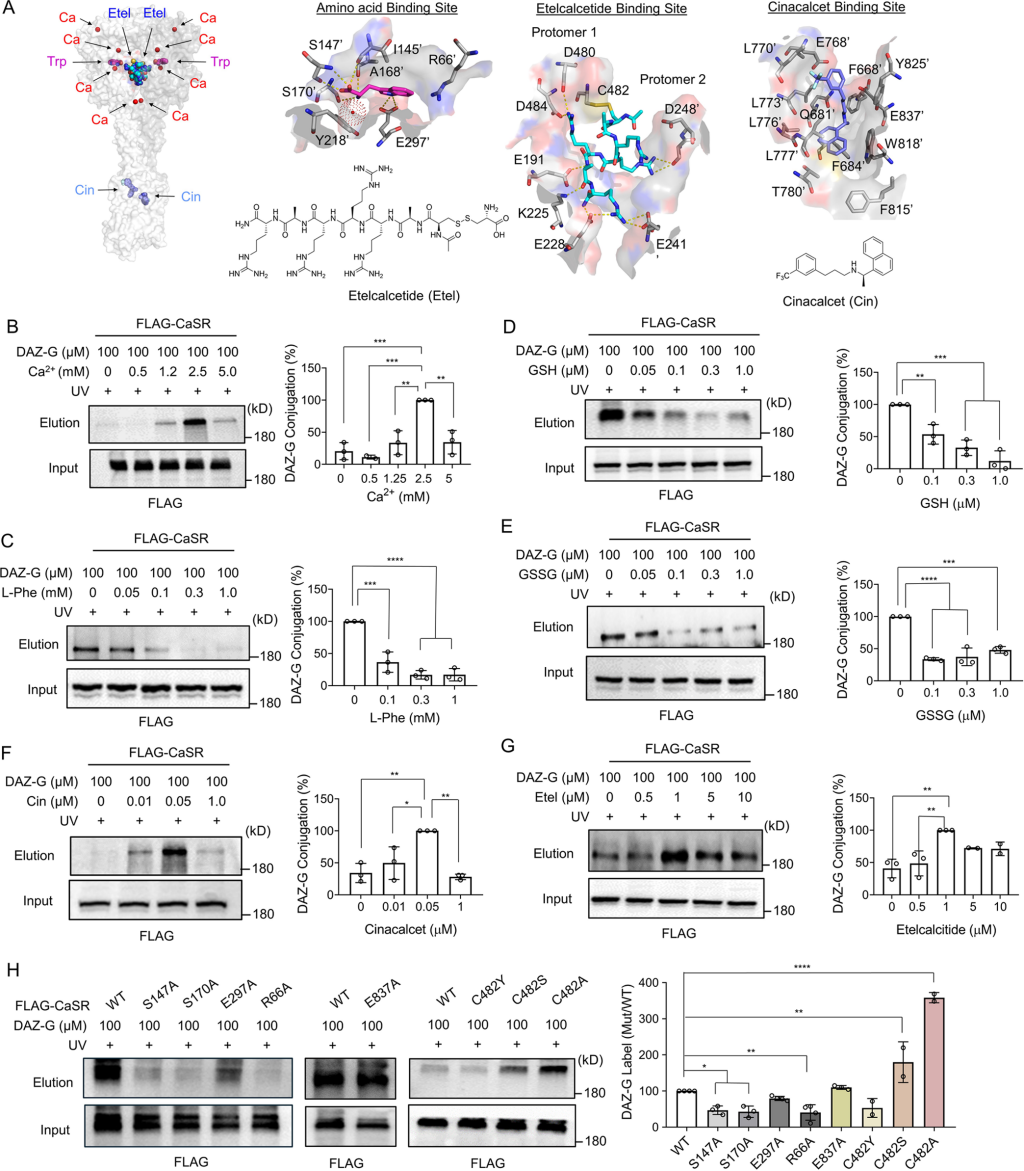
DAZ-G binds to the amino acid binding site in CaSR. (A) Ligand binding sites in CaSR, including the amino acid binding site (ABS), cinacalcet (Cin) binding site (CBS), and etelcalcetide (Etel) binding site (EBS). (B) Ca^2+^_o_-dependent DAZ-G conjugation to CaSR. DAZ-G was incubated with increasing concentrations of Ca^2+^_o_. (C–G) DAZ-G′s binding to CaSR competes with an amino acid, but not with cinacalcet nor etelcalcetide. DAZ-G conjugation to CaSR was monitored in the presence of individual PAM compounds, including l-Phe (C), GSH (D), GSSG (E), cinacalcet (F), and etelcalcetide (G). (H) DAZ-G interacts with residues in the amino acid binding site (ABS). DAZ-G conjugation was compared among CaSR WT and mutants at ABS (S147A, S170A, E297A, R66A), CBS (E837A), and EBS (C482Y, C482S, C482A). DAZ-G and competitors were added to cells in HBSS with Ca^2+^_o_ (2.5 mM) and Mg^2+^_o_ (1.0 mM). Data represent the mean ± SD with representatives from 3 independent experiments. The statistical difference was analyzed by one-way ANOVA with Dunnett’s *posthoc* test (B–H), where *p < 0.05, **p < 0.01, ***p < 0.001, and ****p < 0.0001.

Next, we sought to delineate the binding site of DAZ-G within CaSR. GSH and GSSG, along with all amino acids, are thought to bind the ABS in CaSR, which is positioned in the cleft between LB1 and LB2 (Figures 3A and S4A). In contrast, cinacalcet and etelcalcetide have distinct binding sites (CBS and EBS) in CaSR (Figures 3A and S4A).^16^ First, we evaluated the DAZ-G label on CaSR in the presence of a nominal agonist, Ca^2+^_o_ (0–5 mM) (Figure 3B). Interestingly, DAZ-G conjugation to CaSR depended on Ca^2+^_o_ concentrations, displaying no or weak binding at lower Ca^2+^_o_ (0–0.5 mM, lane 1–2) but apparent conjugation at the physiologic and basal Ca^2+^_o_ (1–1.5 mM) (lane 3). The DAZ-G conjugation was maximal at 2.5 mM Ca^2+^_o_ (lane 4) but decreased at a higher concentration (5 mM) (lane 5). This is consistent with the fact that PAM or GSH is effective only in the presence of Ca^2+^_o_.^21^ Higher Ca^2+^_o_ (0.5–2.5 mM) likely drives CaSR to active states that could enhance the probe conjugation, analogous to the cooperative binding of Ca^2+^_o_ and l-Trp to CaSR.^17,19^ The reduced conjugation at the highest Ca^2+^_o_ (5 mM) may indicate that high Ca^2+^ competes or interferes with the probe conjugation, which may be attributed to the fact that one Ca^2+^ binding site and the ABS are located in the proximity (Figure S4B). Similarly, DAZ-G conjugation to CaSR showed dose dependency with extracellular Mg^2+^ (Mg^2+^_o_) alone or in combination with Ca^2+^_o_ and Mg^2+^_o_ (Figure S2D). Considering the highest DAZ-G conjugation with 2.5 mM Ca^2+^_o_, the subsequent DAZ-G conjugation experiments were conducted in the presence of 2.5 mM Ca^2+^_o_.

Next, we investigated potential competitions of DAZ-G conjugation by representative ligands for distinct binding sites, including Ca^2+^, Phe, GSH, GSSG, cinacalcet, and etelcalcetide, which could indirectly analyze the DAZ-G binding site. As expected, l-Phe, a prototype ligand for ABS, induced concentration-dependent competition to DAZ-G (Figure 3C). Similarly, GSH and GSSG also showed dose-dependent competition (Figure 3D,E). Moreover, glutathione-Cys disulfide (CysSG)^23^ reduced the DAZ-G conjugation to CaSR (Figure S3B). Their competing concentrations are comparable to concentrations of individual compounds for CaSR activation (EC_50_ = 0.3 mM, 0.1 μM, and 0.3 μM for l-Phe, GSH, and GSSG).^21,22^ In contrast, the DAZ-G conjugation to CaSR was not reduced by cinacalcet nor etelcalcetide at concentrations close to their EC_50_ values (0.05 and 0.5 μM, respectively^31,32^) (Figure 3F,G). Instead, the DAZ-G conjugation was unexpectedly increased by cinacalcet or etelcalcetide at certain concentrations (Figure 3F,G). We reasoned that the binding mode of etelcalcetide, which closes two protomers toward the active “closed” state,^16^ could drive the ABS to accommodate DAZ-G more significantly (i.e., etelcalcetide drives CaSR to an active state that increases DAZ-G binding), which is analogous to Ca^2+^_o_-induced higher DAZ-G labeling on CaSR (Figure 3B). Similarly, cinacalcet also caused increased DAZ-G conjugation. Therefore, we suspect that the agonist or PAM binding to CBS or EBS, which induces the active CaSR conformation, would increase the ligand (e.g., DAZ-G) binding in ABS, suggesting their positive cooperative binding. These competition experiments support that DAZ-G binds to the ABS with insignificant binding at the CBS and EBS.

To validate the DAZ-G binding to the ABS, we investigated the DAZ-G conjugation to CaSR mutated at each binding site (Figure 3H). The DAZ-G conjugation to CaSR was significantly reduced with S147A, S170A, and R66A (Figure 3H, lane 1 vs 2, 3, and 5), while less significantly with E297A (Figure 3H, lanes 1 vs 4). S147 and S170 interact with the amino- and carboxylate- groups in Trp, while E297 interacts with the nitrogen amine in the indole ring of Trp (Figures 3A and S4A). R66 is positioned away from the center of ABS (Figure 3A). These analyses suggest that DAZ-G binds to the ABS with similar interactions to Trp (e.g., S147 and S170), but its larger size may render it to interact with R66. In contrast, CaSR E837A, a key residue interacting with cinacalcet (Figure 3A),^16^ did not cause a significant reduction of DAZ-G conjugation versus WT (Figure 3H, lanes, 6 vs 7), supporting that DAZ-G does not interact with the CBS. For mutations in CaSR EBS, C482Y was initially evaluated because C482 is a Cys residue forming a disulfide with etelcalcetide (Figure 3A), and C482Y was previously reported to negate the etelcalcetide-mediated CaSR activation.^24^ Interestingly, we observed the apparent reduction in the DAZ-G conjugation to C482Y (Figure 3H, lanes 8 vs 9), which may suggest the potential DAZ-G binding at the EBS. However, C482S and C482A did not cause the reduction while inducing their enhanced conjugations with DAZ-G (Figure 3H, lanes 8 vs 10 and 11). The diminished or enhanced DAZ-G conjugation to EBS mutants may result from two possibilities: first, C482 mutations to large and small amino acids (i.e., C482Y and C482S/C482A, respectively) may reduce or increase the binding space around EBS, thus reducing and enhancing the DAZ-G′s conjugation at the “EBS,” respectively. Second, these mutations (i.e., C482Y and C482S/C482A) cause the negative or positive cooperativity with the ABS, driving the reduced or increased binding of DAZ-G at the “ABS,” respectively. Notably, the DAZ-G did not reduce its conjugation to CaSR WT in the presence of etelcalcetide that binds to the EBS (Figure 3G). Therefore, we reasoned that the latter possibility is more likely. In addition, the EBS is made of multiple negative charges and binds with positively charged etelcalcetide (Figure 3A), whereas DAZ-G is negatively charged (a similar pI value to GSH). Therefore, we concluded that DAZ-G binds to the ABS versus CBS and EBS.

### DAZ-G Activates CaSR and Increases the Intracellular Calcium Level

**Figure 4:**
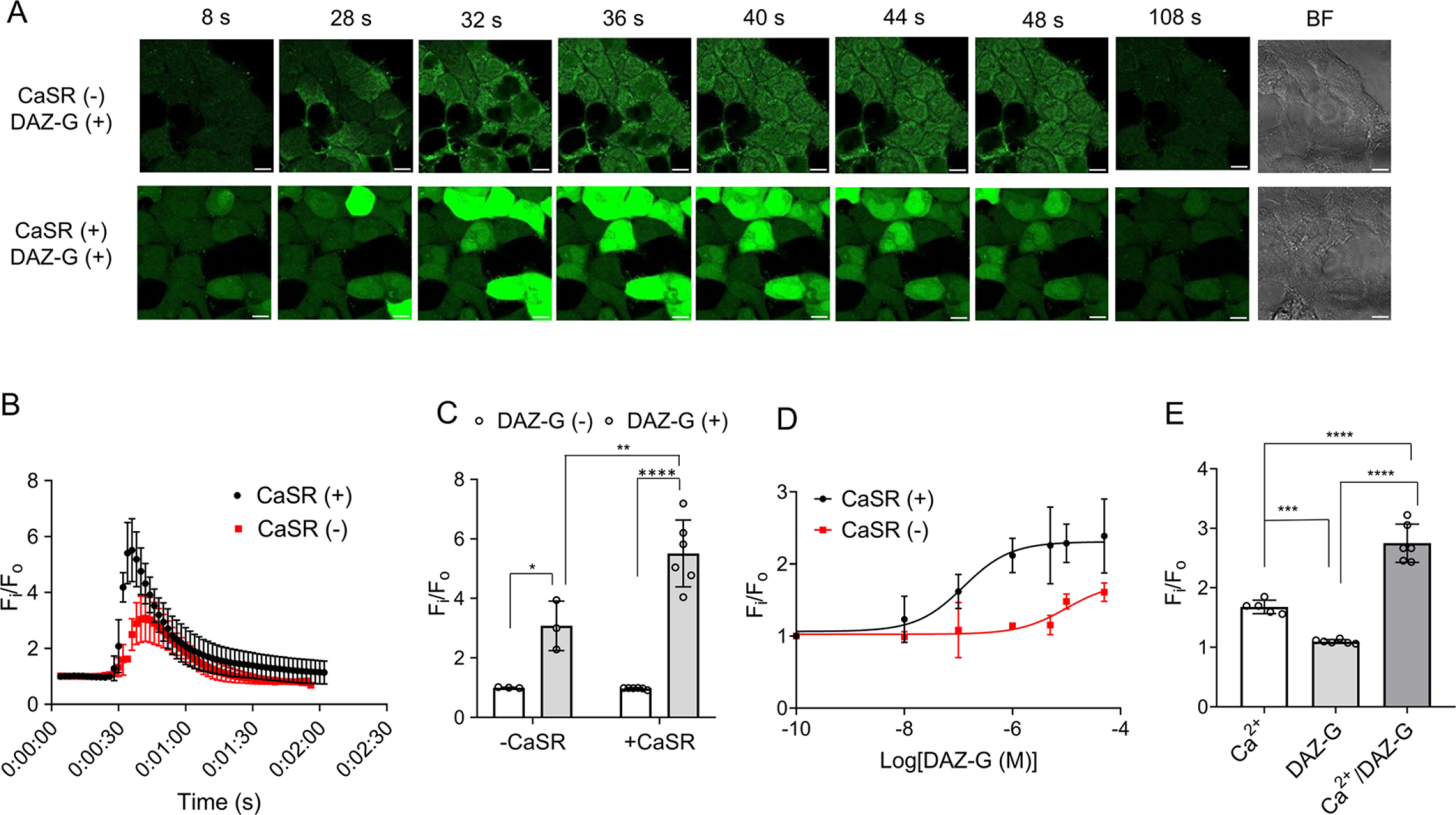
DAZ-G is a positive allosteric modulator of CaSR and increases the intracellular calcium (Ca^2+^_i_) level. The Ca^2+^_i_ increase was monitored using a fluorogenic Ca^2+^ probe (Cal-520). (A) Confocal images of Ca^2+^_i_-induced fluorescence. Ca^2+^_o_ (2.5 mM) and DAZ-G (100 μM) were added at 0 and 28 s, respectively. BF = bright field. A scale bar = 20 μm. (B) Time-dependent fluorescence after (*F*_i_) and before (F_o_) adding DAZ-G. Data show quantification of images in panel (A). (C) Maximum-fold change of fluorescence upon adding DAZ-G. Data are analyzed from panel (B). (D) DAZ-G activates CaSR. A series of DAZ-G concentrations (0–50 μM) were titrated in the presence of Ca^2+^_o_ (0.5 mM) to calculate the potency of DAZ-G (EC_50_ = 115 nM). (E) DAZ-G is a positive allosteric modulator. Fluorescence was measured before (*F*_o_) and after (*F*_i_) adding Ca^2+^ (0.5 mM), DAZ-G (1 μM), or both. Fluorescence in panels (D, E) was measured using the fluorometer. Data represent the mean ± SD with representatives from 3 independent experiments. The statistical difference was analyzed by two-way ANOVA with Sidak’s *posthoc* test (C) or one-way ANOVA with Dunnett’s posthoc test (E), where *p < 0.05, **p < 0.01, ***p < 0.001, and ****p < 0.0001.

As a GPCR, CaSR is primarily coupled to G_i/o_ and G_q/11_ proteins that reduce cyclic adenosine monophosphate (cAMP) levels and activate phospholipase C (PLC), respectively.^15^ The PLC activation generates the secondary messengers, inositol 1,4,5-trisphosphate (IP_3_) and diacylglycerol (DAG), which triggers an intracellular Ca^2+^ (Ca^2+^_i_) release from endoplasmic reticulum (ER) store and protein kinase C (PKC) activation, respectively. With DAZ-G binding to the ABS in CaSR, we evaluated DAZ-G for CaSR activation via the Ca^2+^ flux assay. HEK293 cells ectopically expressing CaSR were incubated with a fluorogenic Ca^2+^ probe (Cal-520). Cells were then incubated with Ca^2+^_o_, followed by the addition of DAZ-G (100 μM) (without UV irradiation), which caused a significant fluorescence spike in a short time (28–90 s, about 5.5-fold peak) (Figure 4A, bottom, and B). In contrast, the fluorescence was less significantly induced upon adding DAZ-G to cells without CaSR expression (about 3.0-fold peak) (Figure 4A, top, and B), supporting the CaSR-mediated Ca^2+^_i_ increase by DAZ-G. However, it is notable that the fluorescence signal was spiked by DAZ-G even in the absence of CaSR (Figure 4B,C), suggesting that DAZ-G may have additional target proteins or mechanisms for Ca^2+^_i_ increase. To measure the potency for CaSR activation, the fluorescence increase was titrated with increasing concentrations of DAZ-G. For this experiment, we generated a HEK293 cell line stably expressing CaSR (HEK293-CaSR) via a CRISPR knock-in system (Figure S2A). DAZ-G in HEK293-CaSR cells (coincubated with 0.5 mM Ca^2+^_o_) induced a 2.3-fold fluorescence increase with relatively high potency for CaSR activation (EC_50_ = 115 nM) (Figure 4D). DAZ-G in cells without CaSR did not increase fluorescence significantly until its high concentrations (>50 μM) (Figure 4D). In addition, DAZ-G did not increase the Ca^2+^_i_-mediated fluorescence without the agonist Ca^2+^_o_ (Figure 4E), supporting that DAZ-G is a PAM for CaSR.

In comparison, we also examined GSSG in the same experiments. GSSG also induced the fluorescence spike upon its incubation only in cells expressing CaSR (Figure S3C,D), albeit the less intense fluorescence increase (about 1.5-fold peak) (Figure S3D) than DAZ-G (5.5-fold) (Figure 4B,C).

### DAZ-G Identifies Allosteric Ligands Interacting with CaSR

**Figure 5:**
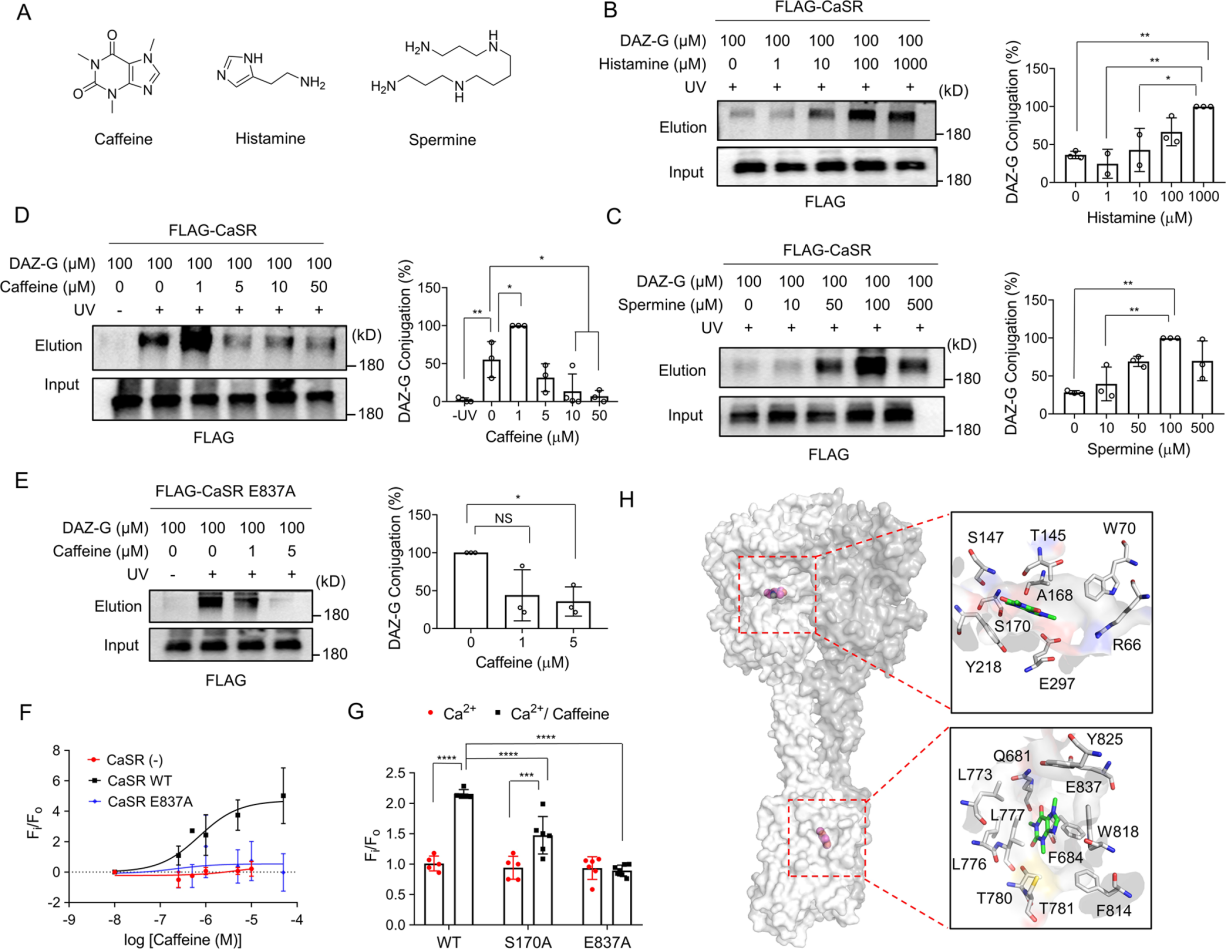
DAZ-G identifies CaSR’s allosteric ligands, finding that caffeine is a positive allosteric modulator of CaSR. (A) Structures of ligands tested for binding to CaSR. (B–D) Evaluation of ligands for potential binding to CaSR. DAZ-G conjugation to CaSR was monitored in the presence of histamine (B), spermine (C), or caffeine (D). DAZ-G and competitors were added to cells with Ca^2+^_o_ (2.5 mM). (E, F) Caffeine interacts with E837 and S170 in CaSR. (E) DAZ-G conjugation to CaSR E837A in the presence of caffeine. (F) Titration of caffeine-induced Ca^2+^_i_ increase in cells expressing CaSR WT or E837A. DAZ-G (0–50 μM) was added with Ca^2+^_o_ (0.5 mM). (G) Caffeine-induced Ca^2+^_i_ increase in cells expressing CaSR WT, S170A, or E837A. Ca^2+^_o_ (0.5 mM) alone or with DAZ-G (5 μM) was added. Fluorescence in panels (F, G) was measured using the fluorometer. (H) Computational docking analysis of caffeine with CaSR structure (PDB: 7M3F). Two proposed binding sites of caffeine in the CaSR structure (left). Two binding modes of caffeine at the ABS and CBS in CaSR (right). Data represent the mean ± SD with representatives from 3 independent experiments. The statistical difference was analyzed by two-way ANOVA with Sidak’s *posthoc* test (G) or one-way ANOVA with Dunnett’s’ *posthoc* test (B–E), where *p < 0.05, **p < 0.01, ***p < 0.001, ****p < 0.0001, and NS = nonsignificant.

One utility of DAZ-G is to identify new ligands binding to CaSR via competition experiments. As a proof of concept, we selected several candidates for CaSR ligands (Figure 5A). Polyamine compounds, including spermine and spermidine, and histamine are increased in asthma patients, contributing to asthmatic symptoms.^7^ These compounds are known for CaSR activation,^26^ but their binding sites in CaSR were not established.

Therefore, in the presence of histamine or spermine (Figure 5A), DAZ-G conjugation to CaSR was monitored in HEK293-CaSR cells. Neither histamine nor spermine reduced the DAZ-G conjugation to CaSR (Figure 5B,C), supporting that histamine and spermine do not bind to the ABS in CaSR. However, interestingly, the DAZ-G conjugation to CaSR increased with histamine or spermine in a range of concentrations close to the compound’s potency (EC_50_ of spermine and histamine = 500 μM^26^ and unreported, respectively) (Figure 5B,C). The increase is reminiscent of CaSR activation by cinacalcet and etelcalcetide, suggesting that spermine and histamine may bind to CBS or EBS, which is partly consistent with a previous finding that spermine binds to a region in or close to 7TM.^33^

### Caffeine is a Positive Allosteric Activator of CaSR

Caffeine is a widely consumed substance. A previous study reported that caffeine negatively regulates PTH release from the parathyroid gland.^34^ However, the report found that caffeine does not induce the Ca^2+^_i_ flux while decreasing cAMP, concluding that caffeine-mediated PTH regulation is likely via caffeine’s well-known targets, adenosine receptors A1 and A2a.^34^ However, the negative regulation of PTH secretion and intracellular cAMP decrease, along with caffeine’s well-known hypercalciuric effects in the human body, correlate with the effects of CaSR activating ligands, prompting us to suspect that caffeine may bind and activate CaSR.

The DAZ-G conjugation experiments show that caffeine in physiological concentrations (0–10 μM, achievable by daily coffee consumption)^35^ competes with DAZ-G binding to CaSR (Figure 5D). Caffeine had a biphasic effect on DAZ-G conjugation to CaSR: DAZ-G binding was increased by caffeine at low concentrations (0–1 μM) (Figure 5D, lanes 2 vs 3) but decreased at higher concentrations (5–50 μM) (Figure 5D, lanes 2 vs 4–6). First, the reduced DAZ-G conjugation at caffeine’s high concentrations (5–50 μM) indicates caffeine directly competing with DAZ-G at the ABS in CaSR. However, the enhanced DAZ-G conjugation to CaSR suggests that caffeine may also bind to CBS or EBS, inducing the positive cooperative binding of DAZ-G at the ABS, as seen with cinacalcet or etelcalcetide (Figure 3F,G). Caffeine’s xanthene structure (Figure 5A) somewhat resembles the cinacalcet’s naphthalene (Figure 3A), suggesting that caffeine could bind to the CBS similarly to cinacalcet. To clarify the binding of caffeine to the CBS, the DAZ-G conjugation to CaSR E837A was monitored in the presence of caffeine. The DAZ-G conjugation to CaSR E837A was not enhanced by caffeine (Figure 5E, lanes 2 vs 3), which contrasts with DAZ-G′s enhanced conjugation to WT upon adding caffeine (Figure 5D, lanes 2 vs 3). Based on these analyses, we conclude that caffeine likely binds to the CBS at lower concentrations (0–1 μM), increasing the DAZ-G conjugation to the ABS. However, caffeine also binds to the ABS at higher concentrations (5–10 μM), eventually decreasing the DAZ-G conjugation to CaSR.

Next, to validate that caffeine is a CaSR activator and primarily binds the CBS and ABS, we evaluated caffeine with the Ca^2+^_i_ flux assay. In contrast to a previous report,^34^ caffeine caused a dose-dependent elevation of Ca^2+^_i_ in cells expressing CaSR WT in the presence of low Ca^2+^_o_ (0.5 mM) with relatively potent activation (EC_50_ = 3.35 μM) (Figure 5F, black). However, such elevation was not seen in cells without expressing CaSR (Figure 5F, red). Caffeine did not induce the Ca^2+^_i_ elevation in cells without Ca^2+^_o_ (Figure S5A), demonstrating that caffeine is a PAM for CaSR. Notably, the caffeine-mediated Ca^2+^_i_ elevation was significantly diminished in cells expressing CaSR E837A (Figure 5F, blue). The caffeine-mediated Ca^2+^_i_ elevation was also reduced in cells expressing S170A (in ABS), albeit not as significantly as E837A (Figures 5G and S5B). Given that both CaSR E837A and S170A are as functional as WT in responding to Ca^2+^_o_,^27^ and considering our DAZ-G binding studies, our data support that caffeine binds to the CBS primarily, while it also binds to the ABS, presumably with the reduced affinity.

Lastly, caffeine’s potential binding modes with CaSR were examined by computational docking analysis (CaSR PDB: 7M3F). Autodock analysis showed caffeine’s potential interactions with residues in the ABS and CBS (Figure 5H). Overall, our data report that caffeine is a potent PAM for CaSR.

## Conclusions

CaSR, belonging to the Class C GPCR, has been investigated for its central role in Ca^2+^ homeostasis and PTH regulation in the parathyroid, kidney, bone, and intestine,^1^ but it is also emerging for its multimodal roles, including asthma, inflammation, and bone metastasis.^7,8,36,37^ Accordingly, diverse activators and inhibitors for CaSR were developed and evaluated in multiple disease models.^15^ In addition to many therapeutic compounds, CaSR is believed to have evolved to sense many endogenous ligands, including metal ions, anions, polycations, amino acids, peptides, and even protein (eosinophil cationic protein associated with asthma).^15,38^ However, ligand binding with CaSR was analyzed mainly by radiolabeled ligand experiments and technically challenging structural biology methods, such as crystallography and cryo-electron microscopy (cryo-EM).^16,19,21,27^ In this report, we designed a photoaffinity-tagged chemical tool (DAZ-G) derived from a CaSR ligand GSH that helps identify and validate CaSR ligands. We demonstrated that DAZ-G binds to the ABS in CaSR, as GSH and many other peptides do, and acts as a potent PAM for CaSR signaling (i.e., Ca^2+^_i_ flux). The photoaffinity-based conjugation of DAZ-G was used to monitor, validate, and discover new ligands for CaSR and their potential interactions at the ABS, CBS, or EBS. One interesting and unexpected feature of DAZ-G is that DAZ-G conjugation to CaSR is not only competed and reduced by ABS ligands but also enhanced by CBS or EBS ligands. This feature helps identify CaSR PAM beyond ABS ligands, although the probe binds to the ABS. This interesting feature is attributed to our observation that the ABS, CBS, and EBS appear to show positive cooperative binding, i.e., one PAM drives CaSR to active conformation, thereby increasing another PAM binding, as seen in the cooperative binding of Ca^2+^_o_ and Trp.^17^ Indeed, recent cryo-EM studies utilized multiple CaSR PAMs that bind to ABS, CBS, or EBS to help determine the active CaSR conformation.^16^ Therefore, the DAZ-G could be an important tool for studying CaSR or identifying new PAM or NAM for CaSR. However, the utility of the DAZ-G probe is currently limited to the analysis of individual PAMs via Western blot analysis. In the future, a fluorophore-conjugated DAZ-G or glutathione derivative could be developed for fluorescent or luminescent assays, such as NanoBRET,^39^ to screen and identify CaSR PAMs or NAMs.

Another important finding in this report is that caffeine is a potent PAM for CaSR. A previous study reported that caffeine does not increase Ca^2+^_i_ in the fluorogenic Ca^2+^_i_ assay.^34^ However, that experiment was conducted using Fura-2. Previously, caffeine was reported to interfere with fura-2 in sensing Ca^2+^_i_,^40,41^ leading us to suspect a false negative result in the previous report.^34^ Indeed, we also found that caffeine-mediated Ca^2+^ elevation was not observed when using the fura-2 Ca^2+^ probe (data not shown). In contrast, our data, such as the DAZ-G competition experiments and the Ca^2+^ elevation assay by an alternative Ca^2+^ probe (Cal-520), demonstrate that caffeine activates CaSR and elevates the Ca^2+^_i_ level. Therefore, our data support the hypothesis that caffeine inhibits PTH secretion in human parathyroid cells^34^ via CaSR. However, it is worth noting that our current study only monitored the Ca^2+^_i_ level as a downstream event of CaSR activation. Therefore, future experiments can use additional readouts, such as TRUPATH biosensors,^42^ to understand the CaSR’s downstream signaling events induced by caffeine and other PAMs.

Caffeine is one of the most consumed plant-derived chemicals and is well known for its effects, such as sleeplessness, restlessness, and excitability, along with dehydration, urination, and palpitation.^43^ These effects are believed to be mediated mainly by antagonism of adenosine receptors A1 and A2a, which are functionally associated with releasing many hormones, including norepinephrine, dopamine, serotonin, acetylcholine, and others.^43^ Although other targets for caffeine were identified, such as phosphodiesterase, GABA receptor, and ryanodine receptor, their relative affinities are low (effective concentrations >200 μM).^43^ In contrast, caffeine activates adenosine receptors with concentrations of 10–50 μM,^43^ plasma concentrations achievable via daily consumption of caffeinated drinks.^35^ Similarly, our data support that caffeine is potent for CaSR activation (EC_50_ = 3.35 μM in 0.5 mM Ca^2+^_i_), leading us to hypothesize that CaSR at many locations throughout the body is likely activated by caffeine consumption. However, caffeine’s efficacy for CaSR activation may need more investigation. Lastly, our findings suggest that caffeine may modulate multiple physiological functions of CaSR, including Ca^2+^ regulation, bone development, osteoporosis, cardiac rhythm, asthma, lactation, and cancer metastasis.^1^ Therefore, our finding warrants additional investigation of CaSR and caffeine to understand caffeine’s impacts on the human body.

## Methods

### Photoaffinity Cross-Linking of DAZ-G with CaSR

HEK293 cells with human calcium-sensing receptor plasmid (pCMV-FLAG-CaSR, OriGene, RC211229) or its mutants were incubated with 100 μM of DAZ-G in 1X Hanks’ Balanced Salt Solution (HBSS, Gibco) containing 2.5 mM calcium chloride (CaCl_2_) and 1.0 mM magnesium chloride (MgCl_2_) for 30 min at 37 °C in the dark, or with the competitor compound. Alternatively, DAZ-G and competitors were added to cells in Dulbecco’s modified Eagle medium (DMEM). After irradiating cells with 365 nm UV light (Spectronics Co.) for 10 min, cells were lysed using RIPA buffer (130 μL). Detailed procedures for the cross-linking, the click reaction with biotin-azide, streptavidin–agarose pull-down, and Western blot analysis are described in the Supporting Information.

### Measurement of Intracellular Calcium Release

HEK293-CaSR stable cells were grown on 13 × 54 mm^2^ coverslips (Chemglass) in a 10 cm cell culture dish. At 80–100% confluency, cells were washed with a calcium assay buffer (20 mM HEPES, 146 mM NaCl, 5 mM KCl, 1 mM MgCl_2_) and loaded with 3 μM Cal-520AM for 1 h at 37 °C in a cell culture incubator. Then, the dye was removed, and the cells were washed with the calcium assay buffer. The Cal-520AM loaded cells were further incubated at 37 °C for 30 min for hydrolysis of the acetoxymethyl ester groups of Cal-520AM inside the cells. The coverslip containing cells was inserted into a quartz cuvette (Fisher, 4 mL volume) containing 1.5 mL calcium assay buffer and a micro stir bar (Cole-Parmer) on the bottom. The cuvette was placed in the fluorescence spectrophotometer (Hitachi F-7100) and exposed to 490 nm excitation wavelength, and the fluorescence emission at 515 nm was measured with 1.6 s time intervals. At a 20-s time point, 30 μL of the ligand (0.5 mM Ca^2+^ or 1 μM DAZ-G with 0.5 mM Ca^2+^) in the calcium assay buffer was injected into the cuvette, and the emitted fluorescence was measured continuously for 3 min. The fluorescence emission intensities before (*F*_o_) and after adding the ligand (*F*_i_) were measured at 10 and 60 s time points, when the fluorescence signals were low (a baseline) and the highest (or plateau), respectively. For EC_50_ measurement, different concentrations of DAZ-G (0–50 μM) or caffeine (0–50 μM) with 0.5 mM Ca^2+^ were added to the cells, and the fluorescence signals were recorded. The fluorescence signals from the intracellular calcium release assay were quantified using FL Solutions software.

### Statistical Analysis

All data are shown with the means ± SD and were statistically analyzed by one-way ANOVA, followed by Dunnett’s *posthoc* test, or two-way ANOVA with Sidak’s *posthoc* test. The value *p* < 0.05 is statistically significant.

## Data Availability

All data supporting the findings of this study are available from the corresponding author upon request.

## Supporting Information Available

DAZ-G synthesis; data for HEK293-CaSR stable cell line and CaSR activation by GSH derivatives; CaSR-caffeine modeling; ^1^H- and ^13^C NMR spectra of DAZ-G; and experimental methods for synthesis of DAZ-G probe; cloning; confocal imaging of calcium release; HEK293-CaSR knock-in cell line generation; and computational analysis (PDF)
