# Supplementary material for: Glutathione-Based Photoaffinity Probe Identifies Caffeine as a Positive Allosteric Modulator of the Calcium-Sensing Receptor
Source: ACS Chem Biol. 2024 Jul 8;19(7):1661–70. doi: 10.1021/acschembio.4c00335 (PMC11267565; doi:10.1021/acschembio.4c00335)
Supplement: Supplementary file 1 — cb4c00335_si_001.pdf [file cb4c00335_si_001.pdf]

Supplementary Information

A Glutathione-based Photoaffinity Probe Identifies Caffeine as a Positive Allosteric Modulator of the Calcium Sensing Receptor

Nadee N. J. Matarage Don,[1] Rayavarapu Padmavathi,[1] Talan D. Khasro,[1] Md. Rumman U. Zaman,[1] Hai-Feng Ji,[1] Jeffrey L. Ram,[2] and Young-Hoon Ahn[1]*

[1]Department of Chemistry, Drexel University, Philadelphia, PA 19104, USA

[2]Department of Physiology, Wayne State University, Detroit, MI 48201, USA

*Corresponding author: Young-Hoon Ahn, ya426@drexel.edu; (215) 895-2666

Supplementary Figures.

A

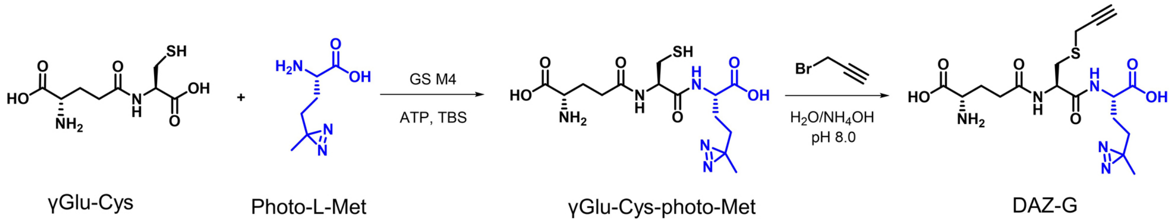

B

GS	Substrate	K_m (mM)	k_{cat} (min^{-1})	k_{cat}/K_m (mM^{-1} min^{-1})
**GS WT***	L-Gly	0.10±0.02	109.50±6.30	1095.00
**GS M4 (F152A/S151G)**	L-Met	0.23±0.20	62.24±0.48	273.22
	L-Photo-Met	0.56±0.95	67.80±26.00	121.07

C

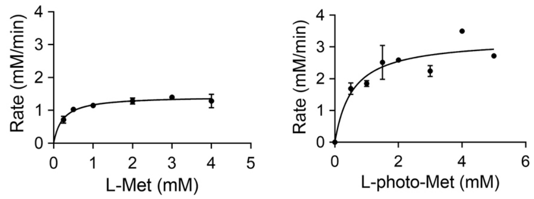

Figure S1. Synthesis of DAZ-G. (A) A synthetic scheme using GS M4 mutant. A glutathione synthetase mutant (GS M4) was used to synthesize γGlu-Cys-photo-Met. (B) Evaluation of GS M4 with photo-Met. For enzymatic biosynthesis, photo-L-Met was tested in enzyme kinetic studies with GS M4, showing that GS M4 can use photo-L-Met as a substrate, albeit with relatively low catalytic efficiencies (ca. 8-9 fold lower than Gly with GS WT). (C) The kinetic curves of GS M4 with substrates. *Kinetic data with WT are obtained from reference.[S1]

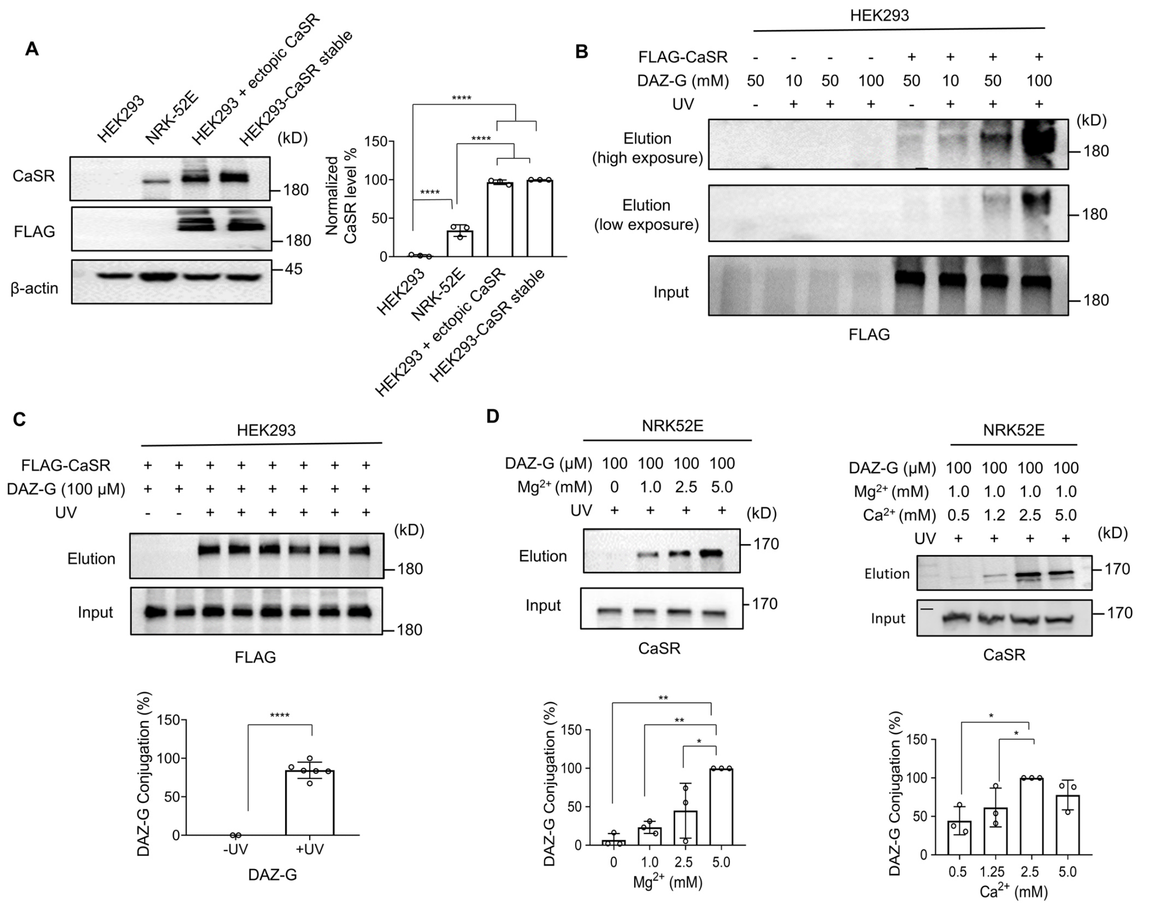

Figure S2. CaSR expression levels in cell lines and DAZ-G's conjugation to CaSR in response to calcium and magnesium. (A) CaSR expression levels in HEK293 cells, NRK52E cell line, HEK293 cells ectopically transfected with CaSR, and HEK293 cells stably expressing FLAG-CaSR (HEK293-CaSR). The HEK293-CaSR cell line was generated using the CRISPR knock-in approach. (B) DAZ-G conjugation experiments in cells with or without expressing FLAG-CaSR. (C) DAZ-G conjugation in cells with or without UV irradiation. (D) DAZ-G conjugation to CaSR in the presence of extracellular Ca^{2+} and Mg^{2+}. After incubation of DAZ-G in cells, the ultraviolet (UV) was irradiated for 10 min. The lysate was subjected to a click reaction with biotin-azide. CaSR was then probed by western blots with CaSR or FLAG antibody before (input) and after (elution) pull-down with streptavidin-agarose. Data represent the mean ± SD with representatives from 3 independent experiments. The statistical difference was analyzed by one-way ANOVA with Dunnett's *post-hoc* test (A, D) or unpaired t-test (C), where $*p < 0.05$, $**p < 0.01$, $***p < 0.001$, $****p < 0.0001$.

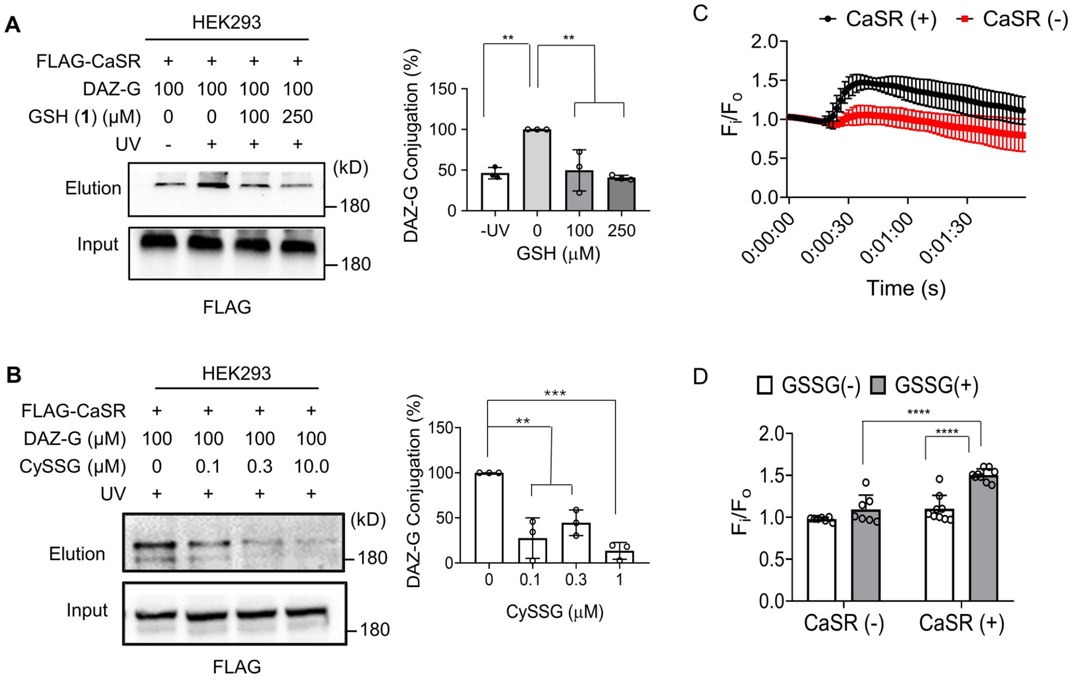

Figure S3. Analysis of glutathione derivatives (GSH, GSSG, and CySSG) for their interaction and activation of CaSR. (A) DAZ-G conjugation to CaSR in the presence of GSH. DAZ-G (100 μM) with different amounts of GSH was added to HEK293 cells ectopically expressing FLAG-CaSR in DMEM containing Ca^{2+}_o (1.8 mM), Mg^{2+}_o (0.8 mM), and amino acids. After UV irradiation for 10 min, cell lysates were collected. After the lysate was subjected to a click reaction with biotin-azide, FLAG-CaSR was probed by western blots before (input) and after (elution) pull-down with streptavidin-agarose. (B) DAZ-G conjugation to CaSR in the presence of CysSG. DAZ-G (100 μM) and CySSG were added to HEK293 cells ectopically expressing FLAG-CaSR in HBSS with Ca^{2+}_o (2.5 mM). The DAZ-G conjugation was analyzed as described in (A). (C-D) GSSG activates CaSR in the Ca^{2+}_i flux assay. (C) Time-dependent fluorescence measurement by confocal imaging after (F_i) over before (F_o) adding GSSG in the presence of Ca^{2+}_o (2.5 mM). (D) The maximum-fold change of fluorescence upon adding GSSG. Data are analyzed from (C). The fold change of fluorescence intensities before (basal) over after (maximum) adding GSSG. Data represent the mean ± SD with representatives from 3 independent experiments. The statistical difference was analyzed by one-way ANOVA with Dunnett's *post-hoc* test (A-B) or two-way ANOVA with Sidak's *post-hoc* test (D), where *$p < 0.05$, **$p < 0.01$, ***$p < 0.001$, ****$p < 0.0001$.

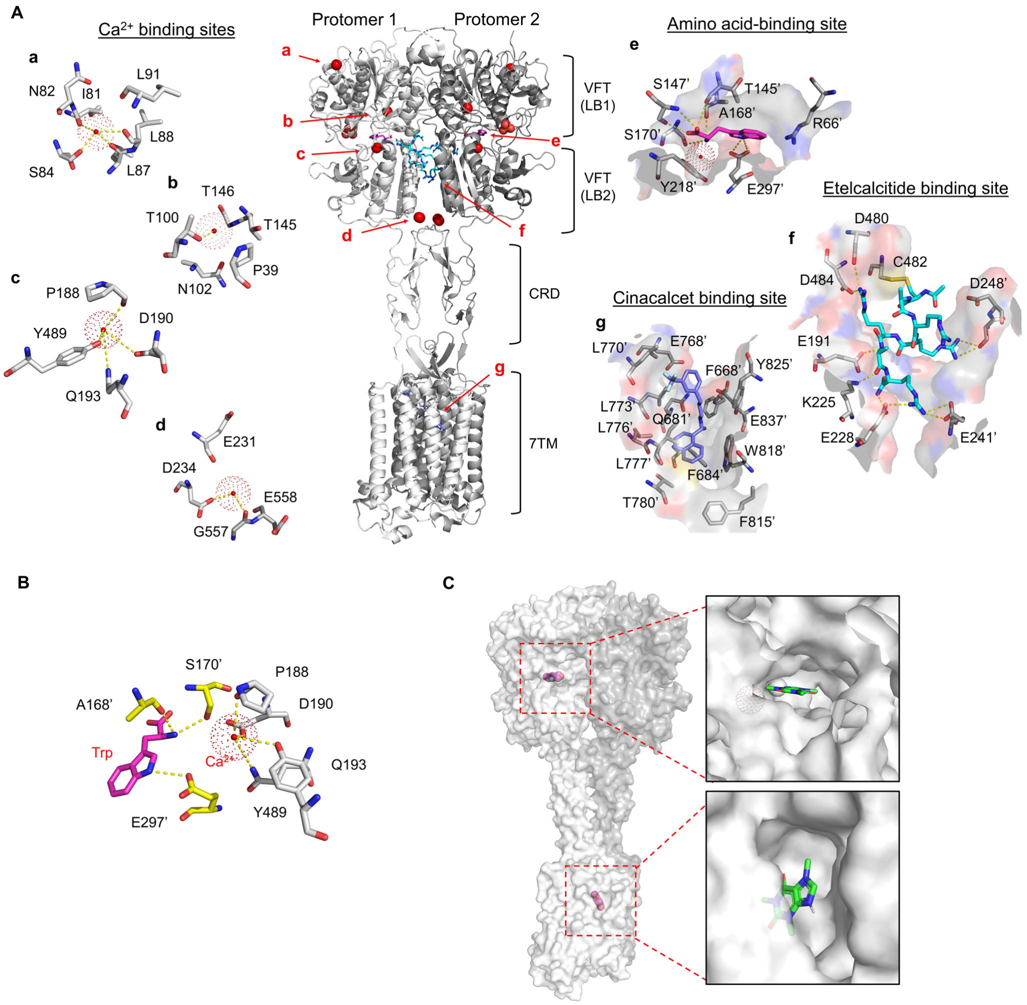

Figure S4. Ligand binding sites in CaSR. (A) Binding sites of calcium and positive allosteric modulators (PAM) in CaSR: four calcium binding sites in the protomers (middle) and the calcium-interacting residues (left). Binding sites of three PAM (middle) and the PAM's interacting residues (right). (B) Tryptophan (Trp) in the amino acid binding site in proximity to a calcium-binding site. A calcium ion is shown as a dot. (C) Caffeine binding sites analyzed by Autodock Vina. The blind docking analysis of caffeine with CaSR (PDB: 7M3F) resulted in 9 modes, among which the strongest binding mode is presented (top right and Figure 5H). The blind docking provided caffeine binding to the ABS only. Thus, additional docking analysis with a grid box (boxing 7TM only) was conducted to find 9 modes of caffeine binding to the CBS, among which the strongest binding mode is presented (bottom right and Figure 5H). A grid box for 7TM: Centre (X: 197.46, Y: 204.78, Z: 243.38) and dimensions in Å (X: 73.76, Y: 63.34, Z:56.59).

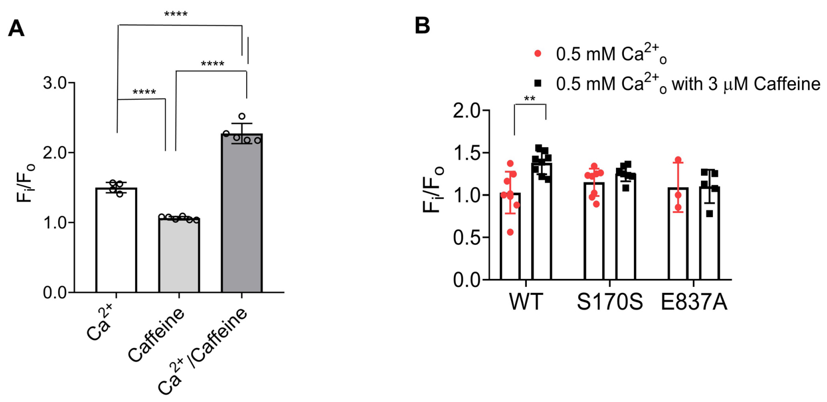

Figure S5. Caffeine is a positive allosteric modulator interacting with S170 and E837 in CaSR. (A) $Ca^{2+}{}_i$-induced fluorescence was measured before (F_o) and after (F_i) the addition of $Ca^{2+}{}_o$ (0.5 mM), caffeine (5 μM), or both. The fluorescence fold change (F_i/F_o) is shown. (B) $Ca^{2+}{}_i$-induced fluorescence upon adding caffeine to HEK293 cells expressing CaSR WT or mutants. The fluorescence increase was measured upon adding caffeine (3 μM) to HEK293 cells in the presence of a low level of $Ca^{2+}{}_o$ (0.5 mM). The fluorescence intensity was normalized by the one in HEK293 expressing CaSR WT upon adding $Ca^{2+}{}_o$ only (bar 1). The statistical difference was analyzed by one-way ANOVA with Dunnett's *post-hoc* test (A) or two-way ANOVA with Sidak's *post-hoc* test (B), where $*p < 0.05$, $**p < 0.01$, $***p < 0.001$, $****p < 0.0001$.

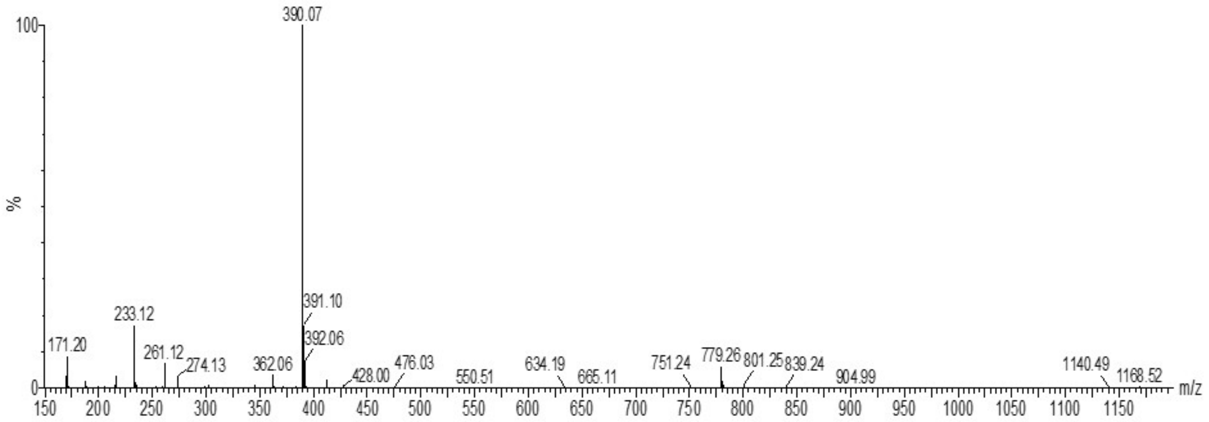

Figure S6. ESI-MS of γ-Glu-Cys-L-photo-Met.

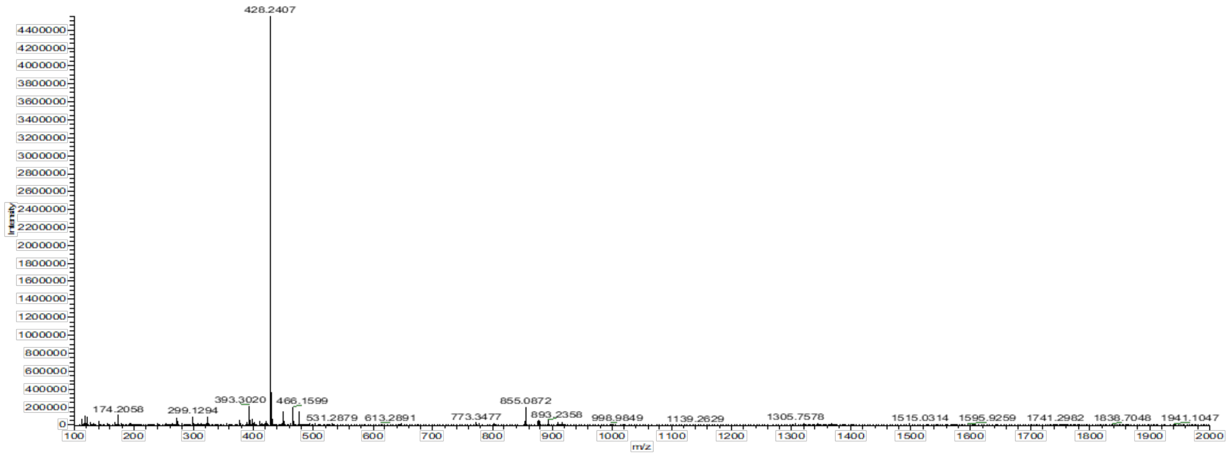

Figure S7. ESI-MS of DAZ-G.

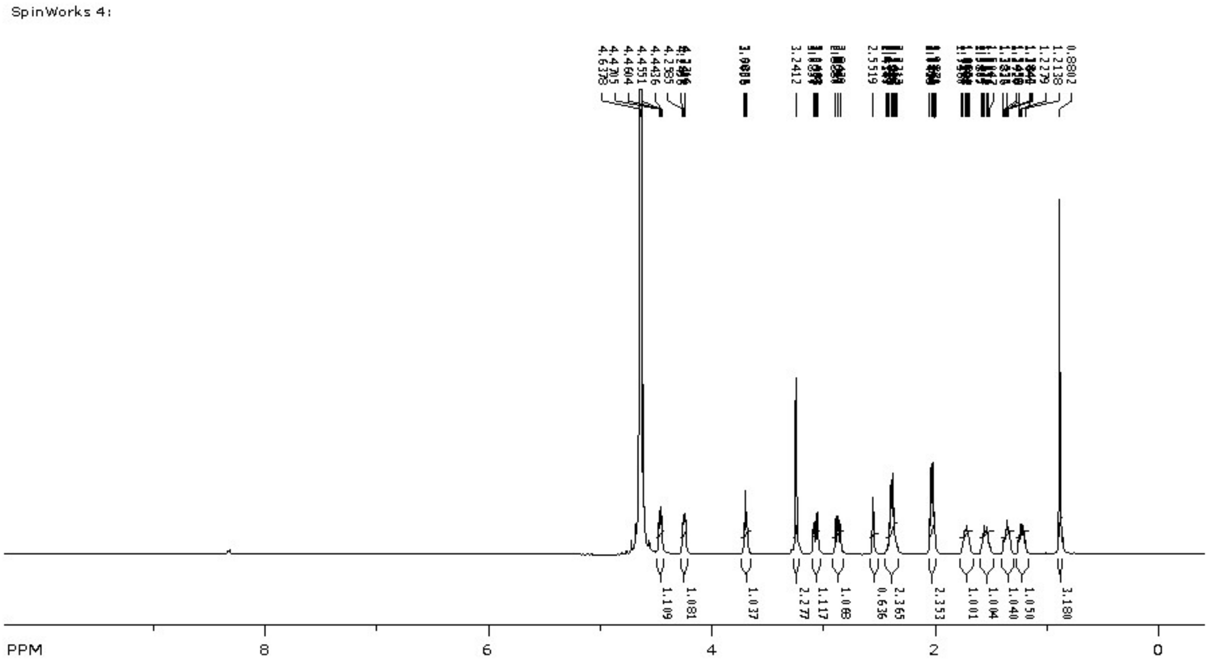

Figure S8. ^{1}H-NMR of DAZ-G.

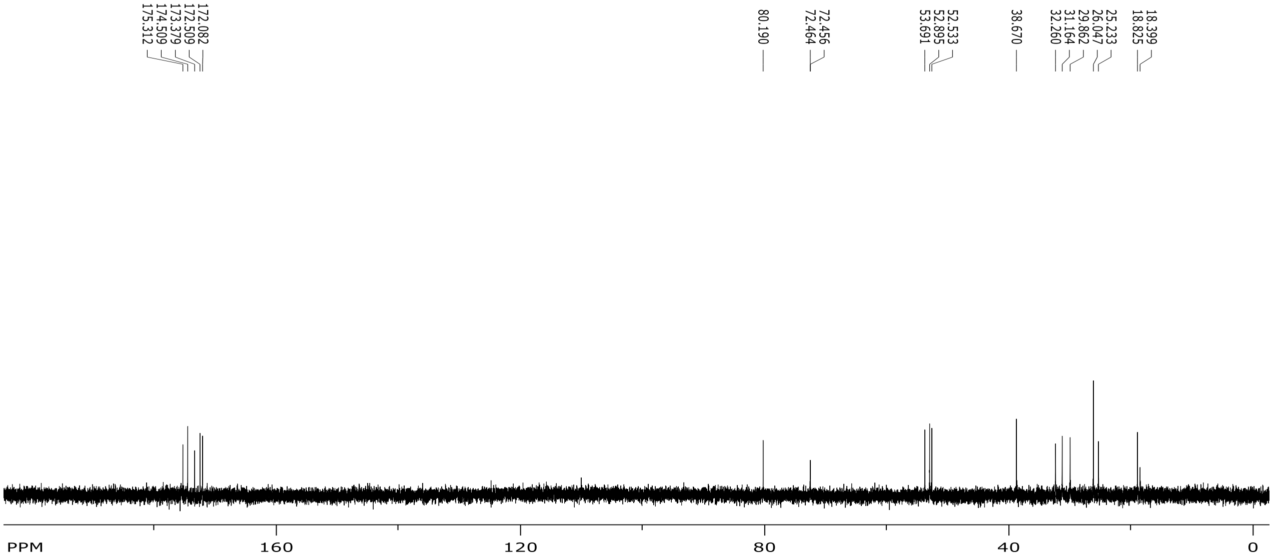

Figure S9. ^{13}C-NMR of DAZ-G.

Supplementary Methods

Synthesis of DAZ-G probe

N5-((S)-1-(((S)-1-carboxy-3-(3-methyl-3H-diazirin-3-yl)propyl)amino)-3-mercapto-1-oxopropan-2-yl)-D-glutamine (γ-Glu-Cys-photo-Met)

γ-glutamyl cysteine (8 mg, 6.4 mM, 1 eq.), L-photo-methionine (Thermo Fisher) (5 mg, 6.4 mM, 1 eq.) and adenosine triphosphate (ATP; Cayman) (35.5 mg, 14 mM, 2.2 eq.) were added to 20 mM Tris HCl buffer (pH 8.0) containing 100 mM sodium chloride (NaCl) and 20 mM magnesium chloride ($MgCl_2$). The glutathione synthetase mutant enzyme[S1] (GS M4; 300 ng, purified in the lab) was added to the reaction mixture and incubated at 37°C for 72 h with rotation. After 72 h, any oxidized product was reduced with 10 mM dithiothreitol (DTT) at room temperature for 1 h. The reaction was quenched by precipitating the GS M4 enzyme with 5% trichloroacetic acid (TCA). The reaction mixture was centrifuged at 4,000 rpm for 10 min and filtered through a syringe filter (Sigma, 0.22 μm). The filtered acidic aqueous solution was injected and separated on reverse-phase C18 column (SunFire, Waters) in HPLC (Acquity, Waters) with a gradient elution of water and acetonitrile (95-5% gradient for 30 min). After lyophilization, the product was obtained as a white solid, giving "γ-Glu-Cys-photo-Met" (8.0 mg, 67%). The purity and mass were confirmed by ESI-MS and LC-MS. Calculated mass: 390.14 (M+H); Found mass: ESI m/z 390.07 (M+H).

N5-((2S)-1-((1-carboxy-3-(3-methyl-3H-diazirin-3-yl)propyl)amino)-1-oxo-3-(prop-2-yn-1-ylthio)propan-2-yl)-D-glutamine (DAZ-G)

γ-Glu-Cys-photo-Met compound (20 mg, 0.514 μmol, 1 eq.) was dissolved in 10 mL of distilled water, and pH was adjusted to 8.0 with ammonium hydroxide (Sigma). Propargyl bromide (Alfa Aesar) (13.7 μL, 15.4 mmol, 3 eq.) was added to the reaction mixture and incubated at room temperature for 2 h with rotation. The solution was injected and separated on reverse-phase C18

column (SunFire, Waters) in HPLC (Acquity, Waters) with a gradient elution of water and acetonitrile (95-5% gradient for 30 min). The product was obtained as a white solid after lyophilization, giving DAZ-G (12.7 mg, 58%). The mass was confirmed by HRMS and LC-MS. Calculated mass: 428.2534 (M+H); Found mass: ESI m/z 428.2407 (M+H). ^{1}H-NMR (500 MHz, D_2O) δ 4.47 - 4.44 (m, 1H), 4.26 - 4.23 (m, 1H), 3.69 (t, J = 6.3 Hz, 1H), 3.24 (s, 2H), 3.07 (dd, J_1 = 14.2 Hz, J_2 = 5.3 Hz, 1H), 2.87 (dd, J_1 = 14.1 Hz, J_2 = 8.5 Hz, 1H), 2.55 (s, 1H), 2.43 - 2.33 (m, 2H), 2.04 - 1.99 (m, 2H), 1.76 - 1.69 (m, 1H), 1.58 - 1.50 (m, 1H), 1.38 - 1.33 (m, 1H), 1.24 - 1.18 (m, 1H), 0.88 (s, 3H); ^{13}C-NMR (125 MHz, D_2O) δ 175.31, 174.51, 172.51, 172.08, 80.19, 72.46, 53.69, 52.90, 52.53, 38.67, 32.26, 31.16, 29.86, 26.05, 25.53, 18.83, 18.40.

Cell culture

Human embryonic kidney cells (HEK293, ATCC) and normal rat kidney-52E cells (NRK52E, ATCC) were maintained in high glucose Dulbecco's Modified Eagle's Medium (DMEM, Cytivia, Cat# SH30022) supplemented with 10% fetal bovine serum (FBS; Cytivia) and 1% penicillin-streptomycin (Pen-strep, Invitrogen) at 37°C in a humidified cell culture incubator. HEK293-CaSR stable cells were maintained in DMEM with 10% FBS, 1% Pen-Strep, and 1 μg/mL puromycin (Gibco) at 37°C in a humidified cell culture incubator.

Photo-affinity crosslinking of DAZ-G with CaSR

HEK293 cells in a 10 cm culture dish were transfected with Myc-DDK-tagged human calcium-sensing receptor plasmid (pCMV-FLAG-CaSR, OriGene, RC211229) or its mutants using lipofectamine 3000 (Thermo Scientific). After 24 h of transfection, cells were split into 6 cm culture dishes. Upon 80-100% confluency, cells were washed with 1X Hanks' Balanced Salt Solution (HBSS; Gibco) and incubated with 100 μM of DAZ-G in HBSS containing 2.5 mM calcium chloride

($CaCl_2$) and 1.0 mM $MgCl_2$ for 30 min at 37°C in the dark. For competition experiments, cells were incubated with 100 μM of DAZ-G and the competitor compound (i.e., GSH, GSSG, or CySSG). DAZ-G was crosslinked with CaSR by irradiating the cells with 365 nm UV light (Spectronics Co.) for 10 min.[S2] Cells were washed with cold HBSS and lysed using RIPA buffer (130 μL) supplemented with a protease inhibitor cocktail (Thermo Scientific). Cell lysates were rotated at 4°C for 45 min and centrifuged at 13,000 rpm for 10 min. The supernatants were collected, and the total protein concentrations of the cell lysates were determined with Bradford assay (Bio-Rad). Cell lysates were stored at -80°C.

Pull-down and western blot analysis of CaSR labeled with DAZ-G

The cell lysates (1 mg protein) from individual samples were precipitated with cold acetone (Fisher) (4 x volume) at -20°C for 1 h. Proteins were pelleted by centrifugation at 6,000 rpm for 3 min. The supernatant was removed, and the protein pellet was air-dried. The proteins were redissolved in a click reaction buffer (144 μL) containing 0.1% SDS, 1X phosphate-buffered saline (PBS), and water. To the solution were added biotin-PEG3-azide (Sigma) (16 μL, 5 mM) and a click mixture (40 μL) consisting of 500 μM THPTA (Click Chemistry Tools), 250 μM $CuSO_4$ (Sigma) and 2.5 mM sodium ascorbate (Sigma). The click reaction was incubated at room temperature for 1 h. Next, the proteins were precipitated with cold acetone (800 μL) at -20°C for 1 h and were pelleted by centrifugation at 6,000 rpm for 3 min. The protein pellets were air-dried and redissolved in 1.2% SDS in PBS (100 μL). For streptavidin enrichment, the protein suspension was added to pre-washed streptavidin agarose beads (Thermo Scientific) in 500 μL of 1x PBS and rotated at 4°C overnight. The following day, the protein-beads mixture was rotated at room temperature for 3 h, and the supernatant was removed by centrifugation at 3,800 rpm for 3 min. The unbound and non-specifically bound proteins were washed with 1X PBS three times. The proteins on streptavidin beads were eluted with SDS-loading buffer (50 μL) containing 100 mM

DTT at 95°C for 10 min. The samples were centrifuged at 13,000 rpm for 10 min and resolved by SDS-PAGE for western blot analysis.

Proteins on the polyacrylamide gel were transferred to the PVDF membrane (Millipore). The membrane was blocked with 5% nonfat milk (MP Biomedicals) in Tris-buffered saline-tween 20 (TBST) containing 50 mM Tris-HCl, 150 mM NaCl and 0.1% Tween 20 (Sigma). Excess milk was washed with TBST. The anti-FLAG primary antibody (Sigma) was dissolved in 5% BSA (Fisher) with TBST (1:1000 dilution) and incubated with the membrane at 4°C overnight. The primary antibody was removed and then incubated with horseradish peroxidase (HRP) linked anti-mouse secondary antibody (Cytivia) (1:2000) in 5% BSA with TBST at room temperature for 1 h. The secondary antibody was removed, and the membrane was washed with TBST. The chemiluminescence signal was developed with HRP substrate (Pierce). The signal was visualized using a gel imager (iBright FL1500). The western blot images were taken with the auto-exposure setup in which all bands show their intensities relative to the strongest band. All the western blot data were quantified using ImageJ software, and the data were analyzed by GraphPad Prism.

Confocal imaging of the intracellular calcium release

HEK293 cells in a 6 cm culture dish were transfected with pCMV-FLAG-CaSR plasmid. After 24 h, cells were split into 35 mm glass bottom culture dishes (MatTek). At 80% confluency, cells were incubated with a calcium assay buffer (20 mM HEPES, 146 mM NaCl, 5 mM KCl, 1 mM $MgCl_2$, and 0.5 mM $CaCl_2$) for 1 h in 37°C cell culture incubator. The cells were loaded with 3 µM Cal-520AM (AAT Bioquest) for 1 h at 37°C. Excess dye was washed, and cells were incubated with the calcium assay buffer containing 2.5 mM $CaCl_2$ for 15 min.[S3] Time-lapse imaging was conducted under the confocal microscope (LSM 700) to visualize the intracellular calcium release response. Fluorescence was

monitored by 490 nm excitation and 515 nm emission wavelengths. Images were captured with 2 s time intervals. At the time point of 28 s, the ligand (i.e., GSSG, or DAZ-G) was added to the cells, and the imaging continued until 3 min. The fluorescence images were quantified using ImageJ software. The data were analyzed by GraphPad Prism.

Generation of CaSR mutants

All the CaSR mutants were generated using site-directed mutagenesis. The mammalian expression vector, pCMV-FLAG-CaSR plasmid (OriGene, Cat. no RC211229) was used as the template, and the primers used for PCR are listed in Table S1.

Table S1. Primer sequences for site-directed mutagenesis of CaSR

CaSR mutant		Primer sequence 5'-3'
CaSR/S147A	F	GGTGGGAGCAACTGGCGCAGGCGTCTCCACGGC
	R	GCCGTGGAGACGCCTGCGCCAGTTGCTCCCACC
CaSR/S170A	F	CAGGTCAGTTATGCCTCCGCCAGCAGACTCCTCA
	R	TGAGGAGTCTGCTGGCGGAGGCATAACTGACCTG
CaSR/E297A	F	ATCTGGCTGGCCAGCGCGGCCTGGGCCAGCTCC
	R	GGAGCTGGCCCAGGCCGCGCTGGCCAGCCAGAT
CaSR/R66A	F	GTGGAATGTATCAGGTATAATTTCGCTGGGTTTCGCTGGTTAC
	R	GTAACCAGCGAAACCCAGCGAAATTATACCTGATACATTCCAC
CaSR/E837A	F	TTTGTCTCTGCCGTAGCGGTGATTGCCATCCTG
	R	CAGGATGGCAATCACCGCTACGGCAGAGACAAA
CaSR/C482Y	F	AGCAGGTGACCTTTGATGAGTATGGTGACCTGGTGGGG

	R	CCCCACCAGGTCACCATACTCATCAAAGGTCACCTGCT
CaSR/C482S	F	CAGGTGACCTTTGATGAGAGTGGTGACCTGGTGGGG
	R	CCCCACCAGGTCACCACTCTCATCAAAGGTCACCTG
CaSR/C482A	F	GGTGACCTTTGATGAGGCTGGTGACCTGGTGGGG
	R	CCCCACCAGGTCACCAGCCTCATCAAAGGTCACC

Generation of HEK293-CaSR knock-in cell line

CRISPR knock-in (KI) of FLAG-CaSR to the pAAVS1 site on human chromosome 19 was conducted using an AAVS1 transgene knock-in kit (OriGene, GE100027). The kit contained two plasmids named pCas-Guide-AAVS1 and pAAVS1-puro-DNR. pCas-Guide-AAVS1 vector contained Cas9 gene and gRNA sequences for AAVS1 site. pAAVS1-puro-DNR contained the AAVS1 site sequence flanked by a puromycin resistance gene and a multi-cloning site for a gene of interest. To clone CaSR gene to pAAVS1-puro-DNR vector, PCR amplification was used with pCMV-FLAG-CaSR as the template, 5'-GGT GGT GCGATCGC ATG GCA TTT TAT AGC TGC TGC TGG-3' as the forward primer with sgf1 as the restriction site, and 5'-GGT GGT GGCCGGCC TTA AAC CTT ATC GTC GTC ATC CTT G-3' as the reverse primer with fse1 as the restriction site. Both the pAAVS1-puro-DNR plasmid and the amplified CaSR gene were double digested with sgf1 and fse1 restriction enzymes (NEB) and ligated using T4 DNA ligase (NEB) to generate pAAVS1-puro-FLAG-CaSR-DNR vector.

HEK293 cells in a 6-well plate were co-transfected with 1 µg of pAAVS1-puro-FLAG-CaSR-DNR vector and 1 µg of pCas-guide-AAVS1 vector using lipofectamine 3000. After the transfection, cells were maintained for three weeks (up to passage number 7). Cells were subjected to puromycin selection (1 µg/mL) for 6-7 days. After puromycin selection, individual cells were isolated into 96-well plates. Monoclonal cell lines were identified and transferred through a 12-well plate to a 6 cm dish. Cells were lysed and subjected to western blot with anti-

FLAG primary antibody and HRP-linked anti-mouse secondary antibody. The cell line with the highest expression of CaSR was identified and maintained as the HEK293-CaSR KI stable cell line.

Computational docking analysis

Docking analysis was performed using PyRx and AutoDock tools 1.5.7 software.[S4-5] Briefly, the receptor file (PDB: 7M3F) was prepared by removing ligands and metal ions using AutoDock tools. Next, hydrogen atoms were added to the receptor, and the charges were assessed using AutoDock tools. The ligand molecule (i.e., caffeine) was drawn in ChemDraw and saved as a mol file. The ligand structure was optimized using Avogadro 1.2.0 software and saved as a PDB file. Ligand optimization includes the assignment of 3D geometry, the addition of hydrogen atoms, and energy minimization. After PDB files of both receptor and ligand molecules were prepared, the docking was performed using PyRx software. The docking data was processed and analyzed using PyMol.

Supplementary References

S1. Samarasinghe, K. T. G.; Godage, D. N. P. M.; VanHecke, G. C.; Ahn, Y. H., Metabolic Synthesis of Clickable Glutathione for Chemoselective Detection of Glutathionylation. *J Am Chem Soc* **2014,** *136*, 11566-11569.

S2. Mackinnon, A. L.; Taunton, J., Target Identification by Diazirine Photo-Cross-linking and Click Chemistry. *Curr Protoc Chem Biol* **2009,** *1*, 55-73.

S3. Kuang, D.; Yao, Y.; Wang, M.; Pattabiraman, N.; Kotra, L. P.; Hampson, D. R., Molecular similarities in the ligand binding pockets of an odorant receptor and the metabotropic glutamate receptors. *J Biol Chem* **2003,** *278*, 42551-42559.

S4. Forli, S.; Huey, R.; Pique, M. E.; Sanner, M. F.; Goodsell, D. S.; Olson, A. J., Computational protein-ligand docking and virtual drug screening with the AutoDock suite. *Nat Protoc* **2016,** *11*, 905-919.

S5. Dallakyan, S.; Olson, A. J., Small-molecule library screening by docking with PyRx. *Methods Mol Biol* **2015,** *1263*, 243-250.
